# Inhibition of the Unfolded Protein Response by Ricin A-Chain Enhances Its Cytotoxicity in Mammalian Cells

**DOI:** 10.3390/toxins3050453

**Published:** 2011-05-10

**Authors:** Chao-Ting Wang, Amanda E. Jetzt, Ju-Shun Cheng, Wendie S. Cohick

**Affiliations:** Department of Animal Sciences, Rutgers, The State University of NJ, School of Environmental and Biological Sciences, New Brunswick, NJ 08901, USA; Email: chaowang@eden.rutgers.edu (C.-T.W.); jetzt@aesop.rutgers.edu (A.E.J.); jushun@aesop.rutgers.edu (J.-S.C.)

**Keywords:** ricin, RTA, ER stress, unfolded protein response, apoptosis, caspase, X-box binding protein1 splicing, IRE1 phosphorylation, eIF2-α phosphorylation, epithelial cells

## Abstract

Ricin is a highly toxic type II ribosome-inactivating protein that has potential as a biochemical weapon and as the toxic component of immunotoxins. The unfolded protein response (UPR) is a survival response that helps cells to recover from endoplasmic reticulum (ER) stress. Failure to recover from ER stress leads to apoptosis. In yeast, ricin-A-chain (RTA), the enzymatic component of ricin, inhibits UPR. Our goals were to determine if RTA inhibits UPR in two epithelial cell lines and if this affects RTA cytotoxicity. RTA alone did not induce UPR. However, RTA inhibited both phosphorylation of inositol-requiring enzyme 1 (IRE1) and splicing of X-box binding protein1 mRNA by the UPR-inducing agent tunicamycin (Tm). The ability of dithiothreitol (DTT) to activate eukaryotic translation initiation factor 2 alpha (eIF2α), a component of the PERK pathway, was also inhibited by RTA. Treatment with RTA in combination with Tm or DTT inhibited protein synthesis more than either agent did alone in one cell line, while caspase cleavage was enhanced by the treatment combination in both cell lines. These data indicate that RTA is more cytotoxic when UPR is inhibited. This ability to inhibit UPR may enhance the potential of RTA as a therapeutic immunotoxin in solid tumors.

## 1. Introduction

Ricin is a glycosylated type II ribosome-inactivating protein (RIP) of 64 kDa that is produced by the castor bean plant *Ricinus communis*. Due to its ease of isolation and extreme toxicity it has been classified by the Centers for Disease Control and Prevention as a category B select agent [1]. There is also interest in using ricin as the toxic component of immunotoxins to target cancer cells [2,3]. Ricin is composed of an enzymatic A-chain (RTA) and a B-chain (RTB) that binds to exposed galactose residues to facilitate uptake by the cell [4]. After entry, ricin undergoes retrograde trafficking to the endoplasmic reticulum (ER), where the disulfide bond between the two subunits is reductively cleaved to release RTA [5,6]. RTA interacts with negatively-charged ER membrane lipids that cause its partial unfolding [7] followed by subsequent transport to the cytosol by the ER-associated degradation (ERAD) pathway that may include interaction with Sec61p, ER degradation enhancing α-mannosidase I-like protein (EDEM) and the Hrd1p ubiquitin ligase complex [8,9,10]. In the cytosol, RTA refolds to a catalytically active form with the assistance of Hsc70 [11]. The *N*-glycosidase activity of RTA depurinates a specific adenine in the sarcin-ricin loop of the 28S ribosomal RNA subunit that is essential for binding of translation elongation factors [12,13], resulting in inhibition of protein synthesis [14].

The ability of type II RIPs, including ricin, to inhibit protein synthesis has been proposed since the 1980s as a major mechanism that underlies their cytotoxicity. However, recent studies have suggested that inhibition of protein synthesis may not be sufficient for RIPs or ribotoxins to induce cell death [15,16]. ER stress is induced by conditions that disrupt the normal ER environment. Since many type II RIPs traffic through the ER, there is interest in determining if ER stress can contribute to their cytotoxicity. ER stress induces the unfolded protein response (UPR), a prosurvival response that serves to restore ER homeostasis during ER stress [17,18]. The UPR increases transcription of ER-localized chaperones and folding enzymes to accelerate the efficiency of protein folding, while general protein translation is inhibited to relieve protein accumulation in the ER lumen. In addition, phospholipid synthesis increases to expand the capacity of the ER while misfolded proteins are retrotranslocated to the proteosome for degradation. However, if the stress is prolonged or these adaptations fail, apoptotic cell death ensues [19].

In mammalian cells, UPR represents a complex signaling network that involves three ER transmembrane proteins: IRE1, PERK and ATF6. Under unstressed conditions, the chaperone protein BiP (*i.e*., Grp78) binds to the luminal domains of these sensors, preventing aggregation. However, during ER stress BiP binds to unfolded proteins, releasing IRE1, PERK and ATF6, which leads to initiation of the UPR [20]. Upon release from BiP, IRE1 homodimerizes and *trans*-autophosphorylates to activate its RNase activity, which cleaves a 26 nucleotide intron from the bZIP-containing transcription factor X-box binding protein (XBP1) to generate a transcriptional activator of a wide array of UPR target genes [17]. PERK also dimerizes and autophosphorylates its kinase domain upon release from BiP, followed by activation of eukaryotic initiation factor 2α (eIF2α), leading to global inhibition of translation and a decreased protein load on the ER [21]. ATF6 translocates to the Golgi apparatus where it is processed by site 1 and site 2 proteases into an active transcription factor that regulates expression of genes involved in ER quality control. Both ricin and shiga toxin have been shown to induce one or more of these branches of the UPR in mammalian cells [22,23]. In contrast, RTA expression in yeast does not induce UPR, but inhibits the UPR elicited by the UPR-inducing agents tunicamycin (Tm) and dithiothreitol (DTT) [24]. The ability of RTA to inhibit the UPR was suggested to contribute to the cytotoxicity of ricin in yeast [24]. The purpose of the present study was to determine if the UPR plays a role in RTA-induced cytotoxicity in mammalian cells. 

## 2. Materials and Methods

### 2.1. Reagents

Thapsigargin (Tg), Tm, dimethyl sulfoxide (DMSO), and RTA were purchased from Sigma-Aldrich. Ricin holotoxin was purchased from Vector Labs. DTT was obtained from AnaSpec Inc. Cell culture media were from Invitrogen and fetal bovine serum was purchased from Atlanta Biologicals.

### 2.2. Cell Culture

The human cervical cancer cell line HeLa was kindly provided by Dr. Tom Obrig (University of Virginia, Charlottesville, VA) and the bovine mammary epithelial cell line MAC-T was established by immortalization with Simian virus 40 large T antigen [25]. Both cell lines were maintained as previously described [26]. For experiments, cells were grown on 60 mm tissue culture plates in complete media containing serum and grown to confluence, washed twice with phosphate buffered saline (PBS) and then changed to serum-free media containing 0.2% bovine serum albumin (BSA) and 30 nM sodium selenite for 2 h. After the serum-free wash, cells were incubated in serum-free media with treatments for various times as indicated in figure legends. 

### 2.3. Western Blotting

Cells were lysed for protein analysis as previously described [26]. Total protein concentration was measured by Bradford protein assay (Bio-Rad Laboratories). Proteins were separated by SDS-PAGE and transferred to 0.45 μm PVDF membrane (Millipore) or nitrocellulose membrane (Bio-Rad). Membranes were blocked in tris buffered saline (TBS) with 0.1% Tween-20 (TBST) in 5% skim milk for 1 h at room temperature and incubated in primary antibodies overnight at 4 °C. After incubation, the membranes were washed three times with TBST for 10 min each and incubated with the appropriate secondary antibody conjugated to horseradish peroxidase (anti-rabbit IgG from GE; anti-mouse IgG from Vector Labs; anti-goat IgG from Santa Cruz) for 1 h at room temperature and then washed three times with TBST. Peroxidase activity was detected by enhanced chemiluminescence substrate (ECL) from Pierce. Total eIF2α antibody was purchased from Santa Cruz; p-eIF2α, total-IRE1, cleaved caspase 3, and total and cleaved caspase 7 antibodies were obtained from Cell Signaling Technology and p-IRE1 antibody was from Novus Biological.

### 2.4. RT-PCR Assay to Detect XBP1 Splicing

Cells were lysed in Trizol (Invitrogen) for RNA analysis according to the manufacturer’s recommendations.

Total RNA was isolated by the Qiagen RNeasy kit. The isolated RNA concentration was measured by the NanoDrop™ 1000 Spectrophotometer (Thermo Scientific) and RNA integrity was examined by agarose gel electrophoresis. To assess XBP1 splicing, total RNA (2 μg) was reverse transcribed using the High Capacity cDNA Reverse Transcription Kit (Applied Biosystems). The PCR primers for human cells were forward primer (F): 5' CCT TGT AGT TGA GAA CCA GG 3'; reverse primer (R): 5' GGG GCT TGG TAT ATA TGT GG 3'. The PCR primers for bovine cells were F: 5' CCT TGT AGT TGA GAA TCA GG 3'; R: 5' GGG GCT TTG TAT ACG TGA 3'. Primers were obtained from Sigma-Aldrich. 10X PCR gold buffer, MgCl_2_, dNTP, and Ampli-Taq Gold were purchased from Applied Biosystems. PCR was performed with an initial denaturation step of 95 °C for 10 min, followed by 35 cycles at 95 °C for 1 min, 50 °C for 1 min, and 72 °C for 1 min, and a final extension step at 72 °C for 10 min. The amplified PCR products were separated by agarose gel electrophoresis on 3% gels and visualized with ethidium bromide.

### 2.5. Quantitative(q) RT-PCR

Primers used for qRT-PCR are listed in Table 1. Each primer set was validated by generating a standard curve from a pool of cDNA with serial dilutions ranging from 1:2 to 1:20,000. The expression of target genes in individual samples was measured using an Applied Biosystems 7300 Real-time PCR system. A dilution of cDNA ranging from 1:4 to 1:20 depending on the gene was amplified in a total reaction volume of 20 μL that included 250 nM forward and reverse primers (5 pmole each) and 10 μL of Power SybrGreen PCR MasterMix (Applied Biosystems). For each experimental sample, values were calculated relative to untreated control samples using the 2^−ΔΔCT^ method with β-actin or cyclophilin as the housekeeping gene.

**Table 1:** Primer design for quantitative RT-PCR.

Gene	Forward Primer (5' to 3')	Reverse Primer (5' to 3')
Human spliced XBP-1	TGCTGAGTCCGCAGCAGG	CGCCAGAATCCATGGGGAGA
Human total XBP-1	GCAAGCGACAGCGCCT	TTTTCAGTTTCCTCCTCAGCG
Human β-actin	AATGTGGCCGAGGACTTTGATTGC	AGGATGGCAAGGGACTTCCTGTAA
Bovine spliced XBP-1	TGCTGAGTCCGCAGCAGG	CATCAGAGTCCATGGGGAGA
Bovine total XBP-1	GCAAGCGACAGCGCCT	TTTTCAGTTTCCTCCGCAGCG
Bovine cyclophilin	GAGCACTGGACAGAAAGGATTTGG	TGAAGTCACCAGCCTGGCACATAA

### 2.6. Protein Synthesis Inhibition

Confluent cells were serum-starved for two h, washed two times with methionine-free media (Invitrogen) and incubated in methionine-free media for 45 min prior to treatment with toxin. Approximately 8 μCi [^35^S] methionine (MP Biomedicals) were added to each sample during the last 30 min of toxin treatment. Cells were washed twice with cold PBS and scraped into cold 5% trichloroacetic acid (TCA). Samples were collected on glass microfiber filters (Whatman) using a vacuum manifold, washed three times with ice-cold 5% TCA and washed two times with cold 95% ethanol. ScintiVerse cocktail (Fisher) was added to each sample and radioactivity was determined by scintillation counting (Beckman Coulter).

### 2.7. Statistical Analysis

Data were analyzed by one-way ANOVA with Bonferroni’s Multiple Comparison *Post-hoc* test or by student’s paired t-test using GraphPad Prism 5 Software.

## 3. Results

### 3.1. RTA Inhibits XBP1 Splicing in Mammalian Cells

**Figure 1:**
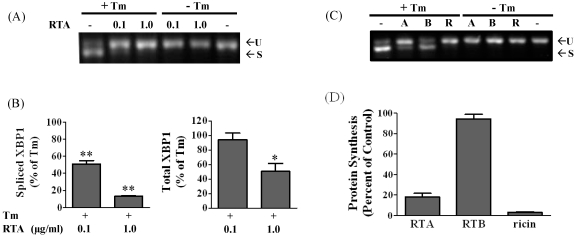
RTA and ricin inhibit tunicamycin (Tm)-induced XBP1 splicing in MAC-T cells. (A) Cells were serum-starved for 2 h prior to treatment ± RTA (0.1 or 1.0 µg/mL) and ± Tm (2.5 µg/mL) for 4 h. Total RNA was analyzed by RT-PCR as described in Materials and Methods. U = unspliced XBP1, S = spliced XBP1; (**B**) qRT-PCR analysis of XBP1 expression. Data were corrected for cyclophilin and presented relative to levels in cells treated with Tm alone. Data were analyzed by one-way ANOVA with Bonferroni’s Multiple Comparison *post-hoc* test. ** *P* < 0.001 and * *P* < 0.01 indicates different from Tm-treated cells. Bars represent mean ± S.E. of four experiments; (C) Cells were treated with 0.1 µg/mL of RTA (A), 1 µg/mL of RTB (B) or 0.01 µg/mL of ricin holotoxin (R) ± 2.5 µg/mL Tm for 4 h and total RNA was analyze by RT-PCR; (**D**) Total protein synthesis was determined by [^35^S]methionine incorporation. MAC-T cells were treated with 0.1 µg/mL RTA, 1 µg/mL RTB or 0.01 µg/mL ricin holotoxin for 4 h. Bars represent mean ± SE of 3 experiments.

XBP1 splicing subsequent to IRE1 phosphorylation is a primary component of the UPR response. Therefore we first examined whether RTA would affect this arm of UPR in the non-transformed mammary epithelial cell line MAC-T. We have previously shown that these cells are sensitive to RTA in terms of depurination, protein synthesis inhibition, and apoptosis within 4 h of treatment [26]. As shown in Figure 1A, only the unspliced form of XBP1 was present in cells treated with RTA (0.1 or 1.0 µg/mL), indicating that RTA did not affect XBP1 splicing. As expected, treatment with Tm, which blocks N-linked glycosylation, caused XBP1 mRNA splicing in MAC-T cells as shown by the decrease in the upper unspliced form and appearance of the smaller spliced form of XBP1. Treatment with 0.1 and 1.0 μg/mL RTA dramatically reversed this effect, with almost complete elimination of the spliced XBP1 band evident by gel electrophoresis. To quantify these changes, a qRT-PCR assay was established. XBP1 splicing was increased 61- to 99-fold by Tm in the three experiments (data not shown). As shown in Figure 1B, RTA treatment inhibited Tm-induced XBP1 splicing by 50 to 87% at concentrations of 0.1 and 1.0 μg/mL, respectively. Total (*i.e.*, spliced plus unspliced) XBP1 mRNA expression was not different between cells treated with Tm alone and those treated with Tm plus 0.1 μg/mL RTA while total XBP1 mRNA levels were 50% lower in Tm-treated cells exposed to the higher concentration of RTA relative to cells treated with Tm alone. These data indicate that the decrease in XBP1 splicing was not a direct reflection of decreases in unspliced XBP1 mRNA levels.

To determine if the holoenzyme acts similarly to RTA, experiments were repeated with ricin holotoxin, RTA and RTB. None of these alone stimulated XBP1 splicing. Ricin and RTA produced similar inhibition of Tm-induced XBP1 splicing, while RTB had no effect on XBP1 splicing induced by Tm (Figure 1C). These concentrations of RTA and ricin inhibited total protein synthesis 82 and 97%, respectively, while RTB had a negligible effect on protein synthesis inhibition (Figure 1D).

**Figure 2:**
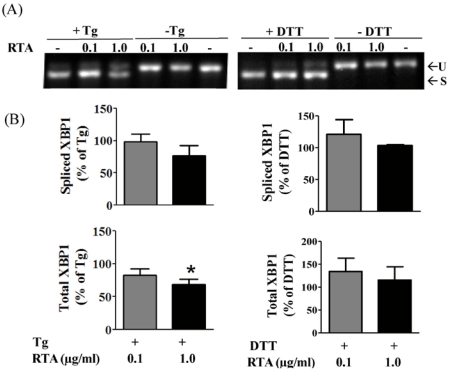
RTA does not inhibit thapsigargin (Tg) or DTT-induced XBP1 splicing in MAC-T cells. Cells were serum-starved for 2 h prior to treatment ± RTA (0.1 and 1.0 µg/mL) and ± Tg (1 µg/mL) or DTT (2 mM) for 4 h. (**A**) Total RNA was analyzed by RT-PCR. U = unspliced XBP1; S = spliced XBP1; (**B**) qRT-PCR analysis of XBP1 expression. Data were corrected for cyclophilin and presented relative to levels in cells treated with Tg or DTT alone. Data were analyzed by one-way ANOVA with Bonferroni’s Multiple Comparison *post-hoc* test. * indicates different from Tg-treated cells (*P* < 0.05). Bars represent mean ± S.E. of four experiments.

UPR is also induced by thapsigargin (Tg), which disrupts calcium homeostasis in the ER, as well as DTT, which reduces protein disulfide bonds. Therefore, we determined if RTA could inhibit the ability of either of these chemicals to induce XBP1 splicing in MAC-T cells. As shown in Figure 2A and B, both Tg and DTT induced XBP1 splicing in MAC-T cells, however, RTA alone did not inhibit XBP1 splicing by either agent. A slight but significant decrease in total XBP1 mRNA expression was observed with 1 µg/mL RTA plus Tg compared to Tg alone, while RTA had no effect on this parameter in DTT treated cells (Figure 2B).

**Figure 3:**
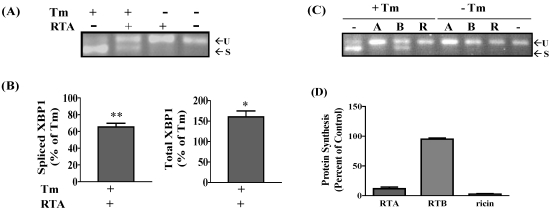
RTA and ricin inhibit tunicamycin (Tm)-induced XBP1 splicing in HeLa cells. Cells were serum-starved for 2 h then treated ± RTA (1.0 µg/mL) and ± Tm (2.5 µg/mL) for 4 h. (**A**) Total RNA was analyzed by RT-PCR. U = unspliced XBP1; S = spliced XBP1; (**B**) qRT-PCR analysis of XBP1 expression corrected for β-actin and presented relative to Tm alone. Data were analyzed by one-way ANOVA with Bonferroni’s Multiple Comparison *post-hoc* test. **P < 0.001 and * *P* < 0.01 indicates different from Tm-treated cells. Bars represent mean ± S.E. of four experiments; (**C**) Cells were treated with 1.0 µg/mL of RTA (A), 1.0 µg/mL of RTB (B) or 0.1 µg/mL of ricin (R) ± 2.5 µg/mL Tm for 4 h; (**D**) Total protein synthesis in cells treated as in (C) for 4 h. Bars represent mean ± SE of 3 experiments.

**Figure 4:**
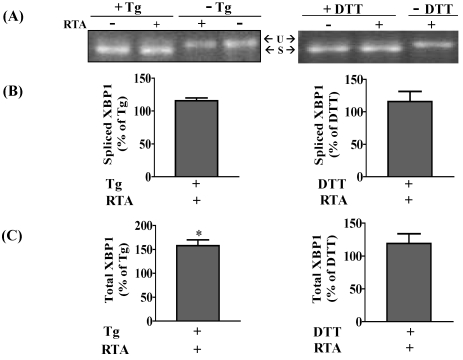
RTA does not affect thapsigargin (Tg)- and DTT-induced XBP1 splicing in HeLa cells. Cells were serum-starved for 2 h prior to treatment ± RTA (1.0 µg/mL) and ± Tg (1.0 µg/mL) for 4 h or ± RTA (1.0 µg/mL) for 4 h and ±DTT (2 mM) added over the last 30 min of treatment. (**A**) Total RNA was analyzed by RT-PCR as described in Materials and Methods. U = unspliced XBP1; S = spliced XBP1; (**B**, **C**) qRT-PCR analysis of XBP1 expression. Data were corrected for β-actin and presented relative to levels in cells treated with Tg or DTT alone. Data were analyzed by paired two-tailed *t*-test. * *P* < 0.05 indicates different from Tg-treated cells. Bars represent mean ± S.E. of four experiments.

In order to determine if the inhibition of Tm-induced XBP1 mRNA splicing by RTA was also observed in transformed cells, the experiments were repeated in HeLa cells, a human cervical carcinoma cell line, which is also responsive to RTA as shown in our previous work [26]. Similar to results obtained with MAC-T cells, RTA alone (1 μg/mL) could not induce XBP-1 mRNA splicing in HeLa cells but did inhibit Tm-induced XBP1 splicing (Figure 3A). This represented an inhibition of 35% when analyzed by qRT-PCR (Figure 3B). The inhibition at 4 h was not due to a decrease in total XBP1 mRNA levels since cells treated with RTA plus Tm expressed more total XBP1 mRNA compared to cells treated with Tm alone (Figure 3B). In addition, ricin holotoxin induced a similar inhibition of Tm-induced XBP1 splicing, while RTB had no effect (Figure 3C). These concentrations of RTA and ricin inhibited total protein synthesis 88 and 95%, respectively, while RTB had a negligible effect on protein synthesis inhibition (Figure 3D). As was observed for MAC-T cells, XBP1 splicing was induced by both Tg and DTT, but RTA had no effect on this response (Figure 4A,B). Interestingly, RTA together with Tg increased total XBP-1 by 50% (Figure 4B).

### 3.2. RTA Inhibits Tm-Induced Phosphorylation of IRE1

**Figure 5:**
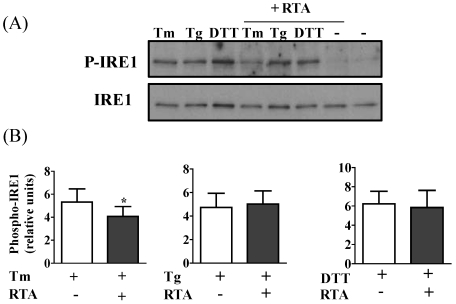
RTA inhibits tunicamycin (Tm)-induced phosphorylation of IRE1 in HeLa cells. Cells were serum-starved for 2 h prior to treatment with RTA (1 μg/mL) ± Tm (2.5 μg/mL) or Tg (1 μg/mL) for 4 h or with RTA (1 μg/mL) for 4 h with DTT (2 mM) added over the last 30 min of treatment. Cell lysates (50 μg) were analyzed by SDS-PAGE. Membranes were immunoblotted with phospho-IRE1 antibody then stripped and reprobed for total IRE1. A representative blot is shown in panel A; (**B**) Data were analyzed by densitometry. Phospho-IRE1 was corrected for loading using total IRE1. Bars represent mean ± S.E. of 5 experiments. Data were analyzed by paired two-tailed *t*-test. * *P* < 0.05 indicates different from Tm alone.

Since RTA inhibited Tm-induced XBP1 mRNA splicing, we looked upstream of XBP1 at IRE1 phosphorylation. As shown in Figure 5A, Tm, Tg, and DTT each induced IRE1 phosphorylation at least 3-fold in HeLa cells. Similar to the results of the XBP1 mRNA splicing assay, RTA alone could not induce IRE1 phosphorylation. However, similar to its ability to decrease Tm-induced XBP1 splicing, treatment with 1 μg/mL RTA decreased Tm-induced IRE1 phosphorylation (Figure 5B). RTA had no effect on DTT- or Tg-induced XBP1 splicing. Similar studies could not be conducted in MAC-T cells since IRE1 antibodies did not recognize the bovine proteins.

### 3.3. RTA Inhibits DTT-Induced Phosphorylation of eIF2α

**Figure 6:**
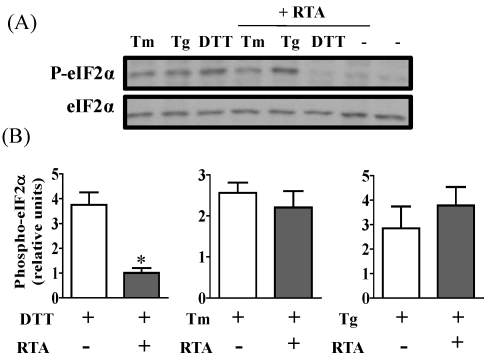
RTA inhibits DTT-induced phosphorylation of eIF2α in HeLa cells. Cells were serum-starved for 2 h prior to treatment with RTA (1 μg/mL) ± Tm (2.5 μg/mL) or Tg (1 μg/mL) for 4 h or with RTA (1 μg/mL) for 4 h with DTT (2 mM) added over the last 30 min of treatment. Total cell lysates (50 μg) were analyzed by SDS-PAGE. Membranes were immunoblotted with phospho-eIF2α antibody then for total eIF2α protein. (**A**) Representative blot is shown; (**B**) Data were quantified by densitometry. Phospho-eIF2-α was corrected for loading using total eIF2-α. Bars represent mean ± S.E. of 3 experiments. * *P* < 0.01 indicates different from DTT alone by paired two-tailed *t*-test.

PERK, another arm of UPR, shares a similar activation mechanism during induction of ER stress. To determine if the PERK pathway was affected by RTA, western blotting of phospho-eIF2α was performed. eIF2α is a direct substrate of phospho-PERK, the activated form of PERK. As expected, phosphorylation of eIF2α in HeLa cells was induced 2- to 4-fold compared to untreated controls by exposure to Tm, Tg or DTT (Figure 6A). RTA alone could not induce eIF2α phosphorylation. When HeLa cells were treated with these UPR-inducing reagents in the presence of RTA, DTT-induced eIF2α phosphorylation was dramatically inhibited by approximately 80% relative to cells treated with DTT alone (Figure 6A,B). However, RTA could not inhibit Tm- and Tg-induced eIF2α phosphorylation (Figure 6C,D). 

### 3.4. Inhibition of ER Stress by RTA Differentially Affects Protein Synthesis Inhibition in MAC-T and HeLa Cells

**Figure 7:**
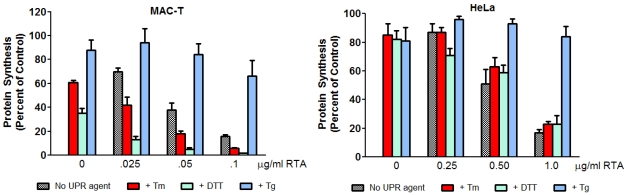
Inhibition of protein synthesis is enhanced in cells treated with both RTA and UPR-inducing agents in MAC-T cells but not in HeLa cells. MAC-T or HeLa cells were serum-starved for 2 h prior to treatment with RTA and Tm (2.5 μg/mL), Tg (1 μg/mL) or DTT (2 mM) for 4 h with the exception that for HeLa cells, DTT was added over the last 30 min of treatment. Total protein synthesis was determined by [^35^S]methionine incorporation. Bars represent mean ± SE of 3 experiments.

Protein synthesis inhibition is often used as a marker of ricin-induced cytotoxicity. To determine if inhibition of UPR by RTA affected this parameter, de novo protein synthesis was measured after 4 h of RTA treatment (Figure 7). MAC-T cells were very sensitive to Tm and DTT in terms of protein synthesis inhibition, with decreases of 39 and 65%, respectively, relative to untreated controls. Cells were less sensitive to Tg, which inhibited protein synthesis less than 20%. When MAC-T cells were treated with Tm or DTT together with RTA, inhibition of protein synthesis was greater than that observed with either agent alone at all three concentrations of RTA examined. Interestingly, when cells were treated with RTA together with Tg, the ability of RTA to inhibit protein synthesis was reduced. In contrast to MAC-T cells, all three UPR-inducing agents decreased protein synthesis approximately 80% in HeLa cells. Overall, RTA had a similar effect on protein synthesis inhibition with or without Tm or DTT. Similar to MAC-T cells, Tg attenuated the ability of RTA to inhibit protein synthesis in HeLa cells.

### 3.5. Inhibition of ER Stress Sensitizes Cells to RTA-Induced Caspase Activation

**Figure 8:**
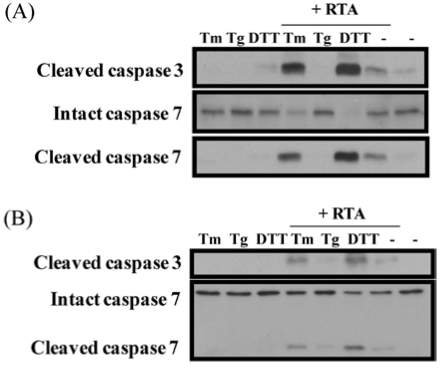
Caspase cleavage is enhanced in cells treated with both RTA and UPR-inducing agents compared to either alone. (**A**) MAC-T cells were serum-starved for 2 h prior to treatment with RTA and Tm (2.5 μg/mL), Tg (1 μg/mL) or DTT (2 mM) for 4 h; (**B**) HeLa cells were serum-starved for 2 h prior to treatment with RTA and Tm (2.5 μg/mL) or Tg (1 μg/mL) for 4 h or RTA for 4 h with DTT (2 mM) added over the last 30 min of treatment. Total cell lysates (30 μg for MAC-T and 50 μg for HeLa) were separated by 15% SDS-PAGE and immunoblotted with cleaved caspase-3, total and cleaved caspase-7 antibodies.

Activation of the UPR pathway represents a survival response that allows the cell to recover homeostasis after an ER overload. Since RTA inhibited the IRE1 pathway induced by Tm and the PERK pathway induced by DTT, we were interested in determining if RTA would activate caspases to a greater degree in the absence of the UPR-survival pathway. As shown in Figure 8, cleavage of caspase-3 and -7 was observed in both MAC-T and HeLa cells treated with RTA. No cleavage was evident when cells were treated with UPR-inducing reagents alone. However, when cells were treated with Tm and DTT in combination with RTA, caspase cleavage was augmented. In contrast, Tg had no effect on caspase cleavage by RTA. These data indicate inhibition of UPR by RTA could make cells more vulnerable to cell death. 

## 4. Discussion

A wide variety of stressors including nutrient deprivation, disruption of calcium homeostasis and hypoxia activate ER stress and elicit the UPR [27,28,29]. RTA unfolds in the ER, causing the cell to perceive it as a misfolded protein that should be targeted to the ER-associated degradation pathway (ERAD) [30]. Therefore, RTA could potentially activate the UPR, which could contribute to its apoptotic effects. However, results of the present study found that RTA alone failed to activate either the IRE1/XBP1 arm or the PERK/eIF2-α branch of the UPR pathway. This agrees with studies in yeast, which also found that RTA alone did not affect HAC1 mRNA splicing, the yeast homologue of XBP1 and the only branch of the UPR pathway that exists in yeast [24]. In contrast with these data, ricin and the RIP riproximin were recently reported to inhibit eIF2-α phosphorylation and to decrease intact ATF-6 protein in two human cancer cell lines, MCF-7 and HCT-11, indicating that these two UPR pathways were induced by toxin treatment [22]. However, similar to the present findings, these investigators also found no XBP-1 splicing in response to toxin exposure. Both of these effects on UPR were observed after 24 h of treatment, in contrast to the present study where cells were treated for only 4 h. Therefore, this may reflect a difference in sensitivity to toxins between different cell types. Interestingly, Shiga toxin, which shares a similar retrograde transport pathway with RTA, has been reported to induce all three branches of the UPR in the human myelogenous leukemia cell line THP-1 [23].

RTA inhibits Tm-induced HAC1 mRNA splicing in yeast [24]. Therefore we investigated whether RTA would have a similar effect on XBP1 splicing in response to ER stress in mammalian cells. We have previously reported that RTA induces apoptosis in both HeLa and MAC-T cells, which represent transformed and non-transformed epithelial cell lines, respectively [26]. RTA inhibited XBP1 mRNA splicing induced by Tm in both cell lines. Since XBP1 splicing is induced by activation of the signaling molecule IRE1 [31,32], we investigated IRE1 phosphorylation and found that RTA also inhibited IRE1 autophosphorylation induced by Tm in HeLa cells. Interestingly, RTA did not inhibit XBP1 splicing or IRE1 phosphorylation induced by DTT or Tg. The ability of RTA to inhibit induction of the PERK arm of UPR, as evidenced by a decrease in eIF2-α phosphorylation, also differed depending on which UPR-inducing agent was used. The explanation for this is unclear, but could relate to different actions of Tm and DTT that result from inhibiting glycosylation versus disulfide bond formation, respectively. In addition, RTA did not inhibit any UPR parameter induced by Tg. During UPR, the accumulation of unfolded proteins in the ER lumen causes the chaperone protein BiP to dissociate from ER-membrane-associated IRE1 and PERK, allowing these molecules to oligomerize and autophosphorylate [33,34]. Therefore, one potential mechanism by which RTA could inhibit phosphorylation would be to reduce general protein synthesis so that BiP fails to dissociate from IRE1 and PERK, which would prevent their activation. However, if this were the mechanism, RTA would be expected to inhibit both signaling pathways in response to Tm and DTT, as well as Tg. The findings that different branches of UPR were inhibited by RTA in Tm-induced ER stress compared to DTT and that no effect was observed on Tg-induced UPR signaling suggest that RTA is not using a general mechanism involving protein synthesis inhibition to inhibit UPR. 

When expressed in yeast, both pre-RTA, which contains the ER localization sequence, and mature RTA, which lacks this sequence and thus fails to enter the ER, inhibit Tm-induced UPR [24]. Based on these data, the authors suggested that RTA inhibits UPR from the cytosolic side of the ER and not the luminal side. Translocation of proteins across the ER membrane requires chaperone proteins, such as BiP and calnexin, which are calcium-dependent [35] and calcium depletion decreases RTA translocation and toxicity in mammalian cells [8]. Therefore, a possible explanation for the failure of RTA to inhibit Tg-induced phosphorylation of IRE1 and/or eIF2-α is that retrotranslocation of RTA from the ER to the cytosol is decreased due to the disruption in ER calcium concentration by Tg. This explanation is supported by the finding that the ability of RTA to inhibit protein synthesis was dramatically reduced in the presence of Tg in both cell lines (Figure 7). These data provide additional support for the idea that the inhibition of UPR by RTA occurs on the cytosolic side of the ER.

When MAC-T cells were treated with Tm or DTT together with RTA, protein synthesis was inhibited to a greater degree that it was with RTA alone, indicating that the two agents are more cytotoxic in combination. Since Tm and DTT each inhibited protein synthesis on their own, this could be an additive effect. Interestingly, in HeLa cells where Tm and DTT had minimal effects on protein synthesis, the addition of either UPR-inducing agent did not alter RTA-induced protein synthesis inhibition. Since UPR is a survival response that pushes the cell toward apoptosis if homeostasis is not restored, we asked the question of whether or not inhibiting the UPR could alter RTA-induced caspase activation as a marker for apoptosis. Treatment of HeLa cells with RTA in combination with Tm or DTT evoked greater caspase cleavage than that observed in response to RTA, Tm, or DTT alone while the ability of RTA to inhibit protein synthesis when UPR was inhibited was not altered. These data indicate that inhibiting the UPR allows RTA to increase cell death through a mechanism that is independent of protein synthesis inhibition. This supports previous findings in yeast using RTA mutants which show that protein synthesis inhibition and cell death can be uncoupled [15]. RTA has been investigated as the toxic component of an immunotoxin that could specifically target cancer cells [2,3,36]. The UPR is activated by hypoxia in solid tumors, resulting in resistance to chemotherapy [37]. Similarly, increases in spliced XBP1 relative to unspliced XBP1 have been shown to correlate with poor prognosis in breast cancer [38] and XBP1 has been proposed as a therapeutic target for solid tumors [39]. Therefore, the ability of RTA to inhibit UPR may make it more potent as a targeted therapy for cancer. This may be particularly useful in cancer cells where UPR is already upregulated by conditions such as hypoxia.

## 5. Conclusions

The present work shows that RTA can inhibit ER stress, leading to an enhancement of its cytotoxicity. ER stress has been shown to be an important component of several disease conditions, including cancer [40]. The ability of RTA to enhance its own cytotoxicity by inhibiting UPR represents a novel mode of action that may enhance its potential as a therapeutic agent in solid tumors.

## References

[1] Audi J., Belson M., Patel M., Schier J., Osterloh J. (2005). Ricin poisoning: A comprehensive review. J. Am. Med. Assoc..

[2] Xu F., Leadon S.A., Yu Y., Boyer C.M., O’briant K., Ward K., Mcwatters A., Zhao X., Bae D.S., Desombre K. (2000). Synergistic interaction between anti-p185HER-2 ricin A chain immunotoxins and radionuclide conjugates for inhibiting growth of ovarian and breast cancer cells that overexpress HER-2. Clin. Cancer Res..

[3] Engert A., Diehl V., Schnell R., Radszuhn A., Hatwig M.T., Drillich S., Schon G., Bohlen H., Tesch H., Hansmann M.L. (1997). A phase-I study of an anti-CD25 ricin A-chain immunotoxin (RFT5-SMPT-dgA) in patients with refractory Hodgkin's lymphoma. Blood.

[4] Olsnes S., Kozlov J.V. (2001). Ricin. Toxicon.

[5] Spooner R.A., Watson P.D., Marsden C.J., Smith D.C., Moore K.A., Cook J.P., Lord J.M., Roberts L.M. (2004). Protein disulphide-isomerase reduces ricin to its A and B chains in the endoplasmic reticulum. Biochem. J..

[6] Bellisola G., Fracasso G., Ippoliti R., Menestrina G., Rosen A., Solda S., Udali S., Tomazzolli R., Tridente G., Colombatti M. (2004). Reductive activation of ricin and ricin A-chain immunotoxins by protein disulfide isomerase and thioredoxin reductase. Biochem. Pharmacol..

[7] Day P.J., Pinheiro T.J., Roberts L.M., Lord J.M. (2002). Binding of ricin A-chain to negatively charged phospholipid vesicles leads to protein structural changes and destabilizes the lipid bilayer. Biochemistry.

[8] Wesche J., Rapak A., Olsnes S. (1999). Dependence of ricin toxicity on translocation of the toxin A-chain from the endoplasmic reticulum to the cytosol. J. Biol. Chem..

[9] Slominska-Wojewodzka M., Gregers T.F., Walchli S., Sandvig K. (2006). EDEM is involved in retrotranslocation of ricin from the endoplasmic reticulum to the cytosol. Mol. Biol. Cell.

[10] Li S., Spooner R.A., Allen S.C., Guise C.P., Ladds G., Schnoder T., Schmitt M.J., Lord J.M., Roberts L.M. (2010). Folding-competent and folding-defective forms of ricin A chain have different fates after retrotranslocation from the endoplasmic reticulum. Mol. Biol. Cell.

[11] Spooner R.A., Hart P.J., Cook J.P., Pietroni P., Rogon C., Hohfeld J., Roberts L.M., Lord J.M. (2008). Cytosolic chaperones influence the fate of a toxin dislocated from the endoplasmic reticulum. Proc. Natl. Acad. Sci. USA.

[12] Moazed D., Robertson J.M., Noller H.F. (1988). Interaction of elongation factors EF-G and EF-Tu with a conserved loop in 23S RNA. Nature.

[13] Endo Y., Tsurugi K. (1987). RNA *N*-glycosidase activity of ricin A-chain. Mechanism of action of the toxic lectin ricin on eukaryotic ribosomes. J. Biol. Chem..

[14] Holmberg L., Nygard O. (1996). Depurination of A4256 in 28 S rRNA by the ribosome-inactivating proteins from barley and ricin results in different ribosome conformations. J. Mol. Biol..

[15] Li X.P., Baricevic M., Saidasan H., Tumer N.E. (2007). Ribosome depurination is not sufficient for ricin-mediated cell death in Saccharomyces cerevisiae. Infect. Immun..

[16] Alford S.C., Pearson J.D., Carette A., Ingham R.J., Howard P.L. (2009). Alpha-sarcin catalytic activity is not required for cytotoxicity. BMC Biochem..

[17] Malhotra J.D., Kaufman R.J. (2007). The endoplasmic reticulum and the unfolded protein response. Semin. Cell. Dev. Biol..

[18] Bernales S., Papa F.R., Walter P. (2006). Intracellular signaling by the unfolded protein response. Annu. Rev. Cell Dev. Biol..

[19] Fribley A., Zhang K., Kaufman R.J. (2009). Regulation of apoptosis by the unfolded protein response. Meth. Mol. Biol..

[20] Rutkowski D.T., Arnold S.M., Miller C.N., Wu J., Li J., Gunnison K.M., Mori K., Sadighi Akha A.A., Raden D., Kaufman R.J. (2006). Adaptation to ER stress is mediated by differential stabilities of pro-survival and pro-apoptotic mRNAs and proteins. PLoS Biol..

[21] Harding H.P., Zhang Y., Bertolotti A., Zeng H., Ron D. (2000). Perk is essential for translational regulation and cell survival during the unfolded protein response. Mol. Cell.

[22] Horrix C., Raviv Z., Flescher E., Voss C., Berger M.R. (2010). Plant ribosome-inactivating proteins type II induce the unfolded protein response in human cancer cells. Cell. Mol. Life Sci..

[23] Lee S.Y., Lee M.S., Cherla R.P., Tesh V.L. (2008). Shiga toxin 1 induces apoptosis through the endoplasmic reticulum stress response in human monocytic cells. Cell. Microbiol..

[24] Parikh B.A., Tortora A., Li X.P., Tumer N.E. (2008). Ricin inhibits activation of the unfolded protein response by preventing splicing of the HAC1 mRNA. J. Biol. Chem..

[25] Huynh H.T., Robitaille G., Turner J.D. (1991). Establishment of bovine mammary epithelial cells (MAC-T): an *in vitro* model for bovine lactation. Exp. Cell Res..

[26] Jetzt A.E., Cheng J.S., Tumer N.E., Cohick W.S. (2009). Ricin A-chain requires c-Jun *N*-terminal kinase to induce apoptosis in nontransformed epithelial cells. Int. J. Biochem. Cell Biol..

[27] Shen X., Zhang K., Kaufman R.J. (2004). The unfolded protein response-a stress signaling pathway of the endoplasmic reticulum. J. Chem. Neuroanat..

[28] Koumenis C., Wouters B.G. (2006). “Translating” tumor hypoxia: unfolded protein response (UPR)-dependent and UPR-independent pathways. Mol. Canc. Res..

[29] Kaufman R.J., Scheuner D., Schroder M., Shen X., Lee K., Liu C.Y., Arnold S.M. (2002). The unfolded protein response in nutrient sensing and differentiation. Nat. Rev. Mol. Cell Biol..

[30] Lord J.M., Roberts L.M., Lencer W.I. (2005). Entry of protein toxins into mammalian cells by crossing the endoplasmic reticulum membrane: Co-opting basic mechanisms of endoplasmic reticulum-associated degradation. Curr. Top. Microbiol. Immunol..

[31] Yoshida H., Matsui T., Yamamoto A., Okada T., Mori K. (2001). XBP1 mRNA is induced by ATF6 and spliced by IRE1 in response to ER stress to produce a highly active transcription factor. Cell.

[32] Calfon M., Zeng H., Urano F., Till J.H., Hubbard S.R., Harding H.P., Clark S.G., Ron D. (2002). IRE1 couples endoplasmic reticulum load to secretory capacity by processing the XBP-1 mRNA. Nature.

[33] Kimata Y., Oikawa D., Shimizu Y., Ishiwata-Kimata Y., Kohno K. (2004). A role for BiP as an adjustor for the endoplasmic reticulum stress-sensing protein Ire1. J. Cell Biol..

[34] Oikawa D., Kimata Y., Kohno K., Iwawaki T. (2009). Activation of mammalian IRE1alpha upon ER stress depends on dissociation of BiP rather than on direct interaction with unfolded proteins. Exp. Cell Res..

[35] Gill D.L., Waldron R.T., Rys-Sikora K.E., Ufret-Vincenty C.A., Graber M.N., Favre C.J., Alfonso A. (1996). Calcium pools, calcium entry, and cell growth. Biosci. Rep..

[36] Zhou X.X., Ji F., Zhao J.L., Cheng L.F., Xu C.F. (2010). Anti-cancer activity of anti-p185HER-2 ricin A chain immunotoxin on gastric cancer cells. J. Gastroenterol. Hepatol..

[37] Feldman D.E., Chauhan V., Koong A.C. (2005). The unfolded protein response: A novel component of the hypoxic stress response in tumors. Mol. Canc. Res..

[38] Davies M.P., Barraclough D.L., Stewart C., Joyce K.A., Eccles R.M., Barraclough R., Rudland P.S., Sibson D.R. (2008). Expression and splicing of the unfolded protein response gene XBP-1 are significantly associated with clinical outcome of endocrine-treated breast cancer. Int. J. Cancer.

[39] Koong A.C., Chauhan V., Romero-Ramirez L. (2006). Targeting XBP-1 as a novel anti-cancer strategy. Cancer Biol. Ther..

[40] Kim I., Xu W., Reed J.C. (2008). Cell death and endoplasmic reticulum stress: Disease relevance and therapeutic opportunities. Nat. Rev. Drug Discov..

